# A Neonate with Subcutaneous Fat Necrosis after Passive Cooling: Does Polycythemia Have an Effect?

**DOI:** 10.1155/2013/254089

**Published:** 2013-07-09

**Authors:** Erhan Calisici, Mehmet Yekta Oncel, Halil Degirmencioglu, Gonca Sandal, Fuat Emre Canpolat, Omer Erdeve, Serife Suna Oguz, Ugur Dilmen

**Affiliations:** ^1^Zekai Tahir Burak Maternity Teaching Hospital, Neonatology, 06230 Ankara, Turkey; ^2^Division of Neonatology, Zekai Tahir Burak Maternity Teaching Hospital, Cebeci, 06230 Ankara, Turkey; ^3^Department of Pediatrics, Faculty of Medicine, Suleyman Demirel University, Isparta, Turkey; ^4^Department of Pediatrics, Faculty of Medicine, Ankara University, 06100 Ankara, Turkey; ^5^Department of Pediatrics, Faculty of Medicine, Yildirim Beyazit University, Ankara, Turkey

## Abstract

Subcutaneous fat necrosis (SCFN) is an inflammatory disorder of adipose tissue. The main risk factors for the development of SCFN are perinatal asphyxia and hypothermia. Presented here is a case of a newborn who developed SCFN in association with polycythemia and hypocalcemia following treatment by passive cooling. Neonates who undergo passive or whole body cooling therapy should be closely monitored for any signs of SCFN.

## 1. Introduction

Subcutaneous fat necrosis (SCFN) of the newborn is a condition characterized by inflammation of subcutaneous fat tissue in term and postterm neonates. The best recognized risk factors for the development of SCFN are asphyxia and hypothermia [1, 2]. The hypoxic conditions created by cold and hypoperfusion trigger the development of granulomatous inflammation of subcutaneous fat tissue followed by necrosis. Other predisposing factors include maternal diabetes, preeclampsia, cocaine abuse or use of calcium channel blockers during pregnancy, birth asphyxia, meconium aspiration, hypothermia, hypoxemia, and hypoglycemia [1].

Presented here is a case of a newborn who required mechanical ventilation immediately after birth due to respiratory failure thought to have developed as a result of meconium aspiration. With a suspicion of birth asphyxia, passive cooling was performed after which SCFN developed 3 days postnatally. To the best of our knowledge, this is the first reported case of SCFN occurring in association with passive cooling.

## 2. Case Report

The patient was a girl born to a 30-year-old mother after 40 weeks of gestation, delivered by Cesarean section. At birth, she weighed 2500 gr. The mother, who was otherwise healthy, had an uneventful pregnancy. On delivery, the amniotic fluid was heavily stained with thick meconium, and the patient's first and fifth minute APGAR scores were 2 and 3, respectively, which prompted intubation and transfer to the neonatal intensive care unit. 

On admission, she was hypoactive and hypotonic, measuring at 47 cm in length (25–50 p) with a head circumference of 33 cm (25–50 p). Results of cord blood gas analysis were consistent with mixed acidosis (pH: 7.01; pCO_2_; 63.4 mmHg; pO_2_: 44.9 mmHg; HCO_3_: 12 mmol/L; BE: −12,1 mmol/L). The patient also did not have any signs of encephalopathy, and findings on amplitude EEG monitorization were normal. A decision to proceed with passive cooling was made as a precaution against hypoxic effects on the brain. This was achieved by first decreasing the set temperature of the incubator and leaving its lid open to maintain a rectal temperature of 34-35°C. 

Hypoglycemia (40 mg/dL), which was encountered within the first few hours after admission, was treated by rapid intravenous infusion of a glucose solution. Blood samples obtained 6 hours after admission revealed CRP and IL6 levels of 29.6 mg/dL and >1000 ng/mL, respectively, for which combination antibiotic therapy with penicillin G and gentamicin was initiated. A hematocrit of 72% also prompted the patient to be treated with partial blood exchange transfusion using saline.

Other laboratory results included a serum AST level of 2110 U/L, ALT of 1033 U/L, CPK of 3887 U/L, LDH of 572 U/L, BUN of 28.9 mg/dL, creatinine of 1.29 mg/dL, and uric acid of 7.94 mg/dL. On day 2 postnatally, serum calcium level was 6.8 mg/dL, while other serum electrolytes were within normal range. Calcium replacement was achieved with calcium gluconate. Blood levels of 25-OH vitamin D, ALP, and parathormone were normal. 

**Figure 1:**
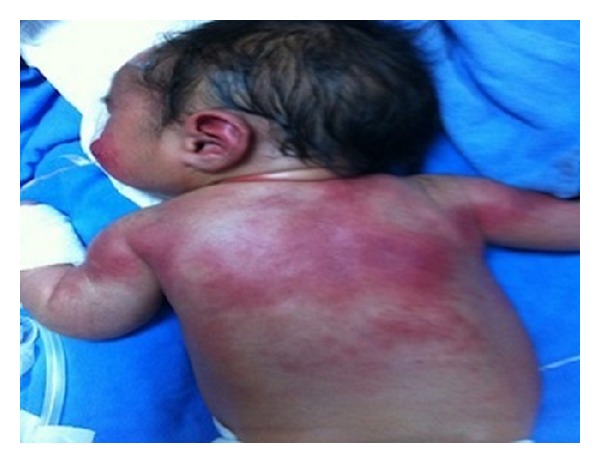
Macroscopic appearance of SCFN in our patient.

**Figure 2:**
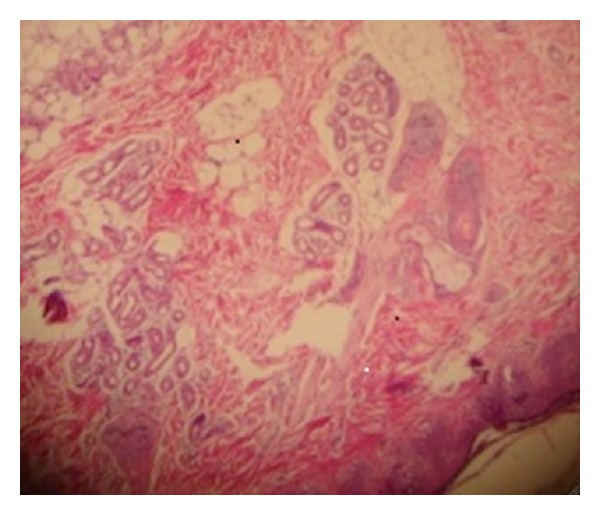
Microscopic appearance of SCFN showing the presence of adipocytes with eosinophilic cytoplasms and histiocytes infiltrating between them.

On the third day postnatally, the patient developed erythema and swelling on her back and over the posterior aspects of her arm, particularly in areas overlying pressure points (Figure 1). A punch biopsy from one of the lesions revealed histopathological findings consistent with SCFN (Figure 2). The patient's lesions gradually improved and the patient was subsequently discharged pending outpatient follow-up.

## 3. Discussion

SCFN is a benign and mostly self-limited condition which usually resolves within a week. Despite recent advances, its etiopathogenesis remains elusive. Previous studies have implicated perinatal asphyxia, hypothermia, meconium aspiration, maternal diabetes mellitus, preeclampsia, sepsis, and birth trauma as risk factors for the development of SCFN [1]. The stress conditions brought about by these factors result in reduced tissue perfusion, and the ensuing hypoxia leads to crystallization of free fatty acids in subcutaneous fat tissue, which is soon followed by tissue necrosis [2]. In a case series by Burden and Krafchik, perinatal asphyxia was the most frequently encountered etiologic factor among 11 patients with SCFN [1]. Several factors may have contributed to the development of SCFN in our patient, including meconium aspiration, respiratory failure, polycythemia, and hypoglycemia. Furthermore, passive cooling may have also been a contributory factor.

To the best of our knowledge, SCFN has not been previously reported in association with passive cooling, while on the other hand a possible association with whole body cooling has been suggested [3, 4]. In our patient, passive cooling is in most likelihood not the sole cause responsible for the development of SCFN, although it may have accelerated the pathogenic process in the presence of other underlying risk factors.

Skin lesions of SCFN may manifest as early as 72 hours, although lesions developing as late as 6 weeks postnatally have also been reported [5]. In our patient, the lesions manifested on the third day of admission. 

Hypoglycemia is another risk factor that has been reported in association with SCFN. However, whether it is a cause or merely a consequence of this condition is still unknown. Although gestational diabetes is an important cause of neonatal hypoglycemia, our patient's mother did not have diabetes. Furthermore, SCFN has been reported to occur in the absence of maternal diabetes [6, 7], and as such it is plausible to suggest that hypoglycemia is a result of the stress posed by hypoxia.

Varan et al. managed to demonstrate that severe anemia in the neonatal period may trigger SCFN by reducing tissue perfusion [8]. Rather than being anemic, our patient had polycythemia which required treatment with partial blood exchange transfusion. We believe that, by affecting tissue perfusion, hypoxia and hypothermia were both primarily responsible for the development of SCFN in our patient. We did not encounter a case of SCFN in association with polycythemia in the literature. 

Complications of SCFN include hypercalcemia, pain, dyslipidemia, renal failure, and late subcutaneous atrophy [9]. Despite being a rare complication, hypercalcemia has a negative impact on morbidity and mortality. Research has shown hypercalcemia to develop as a result of increased prostaglandin activity, release of calcium from necrotic fat tissue, and increased secretion of 1,2-dihydroxy vitamin D from macrophages. Periodic evaluation of serum calcium levels is therefore recommended in such patients. In contrast, our patient developed hypocalcemia on the second day postnatally which required treatment. In a previous report on hypocalcemia occurring in association with SCFN, pseudohypoparathyroidism was identified as the underlying cause [10]. In our patient, however, parathormone, 25-OH vitamin D, and ALP levels were normal.

 SCFN is a commonly encountered problem in newborns who suffer hypoxia. Such patients mostly require follow-up in the intensive care setting where they are exposed to extensive therapeutic procedures and invasive interventions. SCFN is also known to develop on areas overlying pressure points, which highlights the importance of nurse care. Appropriate mobilization and frequent changes in position are among the measurements that could be implemented to preserve tissue perfusion and therefore decrease the likelihood of a patient developing SCFN. 

SCFN is a complication that should come to mind in newborns with birth hypoxia, and the presence of hypothermia and polycythemia may accelerate the pathogenic process. In neonates hospitalized for perinatal asphyxia, special care should be taken to avoid therapeutic hypothermia unless absolutely indicated, while also stressing the importance of nurse care for preventing the development of SCFN.
